# Problems in Diagnosis and Treatment of Soleus Muscle Injuries—Narrative Review and Case Report

**DOI:** 10.3390/jcm14061955

**Published:** 2025-03-13

**Authors:** Robert Trybulski, Kamil Gałęziok, Filip Matuszczyk, Tomasz Halski, Jarosław Muracki

**Affiliations:** 1 Medical Department, The Wojciech Korfanty Upper Silesian Academy, 40-659 Katowice, Poland; 2 Provita Medical Center, 44-240 Żory, Poland; 3 Faculty of Physical Education and Physiotherapy, Opole University of Technology, 46-020 Opole, Poland; 4 Institute of Physical Culture Sciences, Department of Physical Culture and Health, University of Szczecin, 70-453 Szczecin, Poland

**Keywords:** calf muscle rupture, triceps surae, post-traumatic physiotherapy, sports medicine

## Abstract

Injuries to the soleus muscle are often unrecognized, which increases the risk of complete tearing. Consequently, it results in the need for a long break in sports. This is mainly because the soleus muscle is complex, and the clinical signs of injury are difficult to capture, which can mimic Achilles tendinopathy and tennis player’s calves. This muscle has a complex connective tissue structure with three intramuscular tendons, which makes it challenging to interpret pathological muscle conditions. Injuries to the soleus muscle can be acute or chronic and are usually considered to be a minor discomfort by both the patient and the sports medicine physician, leading to a relatively quick return to sports activity with a high risk of re-injury. This narrative literature review aims to explore the diagnostic challenges and treatment failures associated with soleus muscle injuries, highlighting the critical lack of standardized protocols and a comprehensive understanding of the nuances of these injuries, which requires the collection of qualitative data from clinical case studies, quantitative data from imaging studies and rehabilitation outcomes, and expert opinion to formulate evidence-based guidelines to improve patient management. Calf muscle pain symptoms should not be ignored because the injury may become chronic, and the lack of treatment adequate to the actual cause of the pain increases the risk of the injury deepening, including complete rupture. High-resolution ultrasonography and magnetic resonance imaging are recommended methods for differentially diagnosing soleus muscle injury in conjunction with physical examination to make a precise and reliable diagnosis. A soleus muscle injury case report and a comprehensive proposal for conservative treatment supplement our literature review.

## 1. Introduction

While not as commonly discussed as other musculoskeletal injuries in athletic populations, soleus muscle injuries have emerged as a significant concern in sports medicine and rehabilitation [1]. Historically, the soleus muscle, which is located beneath the gastrocnemius in the calf region, has been overshadowed by more prominent muscle injuries, specifically those involving the hamstrings, quadriceps, and gastrocnemius [2]. This disparity is alarming given the soleus’s essential role in not only athletic performance, particularly in activities involving running, jumping, and other dynamic movements, but also in crucial daily functions such as walking and maintaining postural stability [3]. Despite the appellation of “tennis leg” to calf injuries, a study found that only about 16% of athletes with calf injuries are among tennis players. Another study showed that this injury includes 4% of all tennis-related injuries [4].

The theoretical underpinnings for understanding soleus injuries stem from biomechanics and muscle physiology, emphasizing the necessity for targeted research focused on this underappreciated muscle. Despite its critical role, there needs to be more consensus regarding the diagnostic criteria and management strategies for soleus injuries, which complicates treatment and recovery protocols. Moreover, the current body of literature often treats muscle injuries in a generalized context, consolidating various lower extremity injuries without distinguishing between their specific characteristics and treatment needs. Applying generalized treatment strategies can lead to incomplete recovery or even exacerbation of the injury, underscoring the importance of establishing a more refined understanding of soleus muscle injuries [5]. Critical debates within the field focus on the adequacy of existing diagnostic tools, the variability in treatment modalities, and a general lack of standardized rehabilitation protocols.

These issues highlight the pressing necessity for a more nuanced approach that considers the particular complexities of the soleus muscle. This narrative review centers on the investigative need to elucidate the diagnostic challenges and treatment inefficacies associated with soleus muscle injuries [6]. The central question guiding this research is: How can the diagnostic and treatment strategies for soleus muscle injuries be systematically improved, given the current lack of standardized protocols and comprehensive understanding of the injury’s nuances? This inquiry arises from observing a significant gap in the extant scientific literature. There are no well-established, evidence-based medical procedures for differential diagnosis, examination, and treatment of soleus muscle injuries. The diagnostic criteria and treatment strategies for soleus injuries often need to be more adequately detailed, rendering healthcare professionals without clear guidelines for effective patient management. Despite increasing recognition of the soleus muscle’s importance, intervention strategies remain reactive rather than preventive, resulting in prolonged recovery times and increased incidence of recurrent injuries. By interrogating the origins of this problem and analyzing its implications, this study aims to contribute significantly to the field by developing a structured framework for addressing these diagnostic and treatment challenges.

As participation in sports continues to increase, particularly among adolescents and recreational athletes, understanding the unique challenges associated with soleus injuries becomes crucial. Furthermore, effective treatment of these injuries can impact injury recurrence rates, thereby contributing to the overall longevity of an athlete’s career and improving their quality of life. The objectives of this study were to conduct a comprehensive literature review to compile existing diagnostic techniques for soleus injuries in the clinical setting. Second, the study aims to collect and analyze qualitative data from clinical case studies and quantitative data from imaging and rehabilitation outcomes. Finally, the study aims to synthesize expert opinions to develop evidence-based guidelines that better inform clinicians about effective treatment paths.

### 1.1. Muscle Anatomy

The soleus muscle is responsible for plantar flexion of the foot and is a single-joint muscle with a predominance of slow-twitch fibers (type I muscle fibers) [7]. It is vital in maintaining posture and other low-energy activities such as walking and endurance sports. The soleus muscle constitutes 70% of the total muscle surface of the posterior leg, which has two compartments: deep and superficial [8]. It is located deep between the heads of the gastrocnemius, with its proximal origin in the tibia and fibula, and distally joins the gastrocnemius muscle to form the Achilles tendon 8–10 cm above its insertion on the calcaneus [1]. The soleus muscle is supplied by the posterior tibial artery and the peroneal artery, while innervation comes from branches of the tibial nerve [7].

There are significant anatomical anomalies of this muscle. The accessory soleus muscle is a rare anatomical variant that can be asymptomatic or cause pain and swelling in the lower leg, and 20% of the population is missing it. Additional muscle bands can occur (very rarely) and can be double muscles [9].

The soleus muscle has two proximal origins, one peroneal and one tibial. The peroneal origin, which is superior to the tibia, originates from a thick band of tendon in the posterior sector of the head of the fibula and from aponeurotic fibers from the lateral border of the fibula and the posterior intermuscular septum, which separates the soleus from the peroneus longus. The tibial bundle originates from a bony prominence on the posterior surface of the tibia, called the soleus line, and from the medial border of the tibia [6].

The tendons within the soleus muscle are commonly used to define the site of injury in soleus strains. Strains within the soleus muscle often involve the musculotendinous (MTJ) or myofascial (MFJ) junctions, although the spatial relationships between these tendon structures need to be better understood [10]. There are few reports of quantitative measurements of the three-dimensional architecture of the soleus muscle in vivo, in which this muscle exhibits five morphological variants: (1) Bipennate-Midline; (2) Bipennate-Medial Deviation; (3) Bipennate-Lateral Deviation; (4) Unipennate; (5) Hypopennate [10]. The findings suggest that the interconnections between the medial and lateral anterior apertures and the corresponding MTJ and MFJ may influence injury patterns in the soleus muscle. This highlights the need for a deeper understanding of connective tissue architecture in muscle injury [11]. The anatomical changes identified in this study are essential for interpreting diagnostic imaging. Awareness of these changes may help better understand muscle changes and injuries, which is particularly important for clinicians and radiologists.

Cadaveric studies using microdissection techniques and magnetic resonance imaging have shown that the soleus muscle has the largest physiological cross-sectional area of all human lower limb muscles [12]. The fascicles in each compartment have distinctly different orientations. Still, similar lengths make it compartmentalized: it consists of a posterior unipinnate wrapped around the anterior bipinnate radially [12]. The proximal region of the muscle is unipinnate, while the middle area has a radially bipinnate semicylindrical structure, and the distal region is tetrapinnate. Tissue velocity mapping shows that the regions of highest velocity coincide with aponeuroses connected to the Achilles tendon [13]. These are located on the anterior and posterior surfaces of the muscle. The lowest velocities coincide with aponeuroses connected to the muscle’s origin and are usually located intramuscularly. In vivo, the magnetic resonance imaging (MRI) measurements of tissue velocity during isometric contractions (20% and 40% of maximal voluntary contractions) revealed a similarly complex 3D distribution of tissue motions [14]. The study results show a strong relationship between the complex three-dimensional structure of the muscle–tendon system and the intramuscular tissue velocity distribution during isometric contractions in vivo [15].

### 1.2. Causes of Injuries

Although the soleus muscle covers a large area of the posterior leg, its injuries are an unexpected cause of pain and functional deficit, and symptoms are usually attributed exclusively to the gastrocnemius muscle [1]. Fundamental causes are anatomical vulnerabilities that generate stress, especially at the musculoskeletal junctions. Proximal medial tendinous sprains account for 56.4% of soleus injuries [11]. Another group of factors is repetitive activities, especially in sports, which can lead to chronic injuries due to muscle fatigue, especially with a flexed knee [1]. The sudden force the muscle generates quickly can cause acute injury. Minor, challenging-to-diagnose ultrasound injuries to the soleus muscle are often underestimated and confused with gastrocnemius strains or asymptomatic Achilles tendinopathy, complicating treatment and exposing the risk of a more significant injury [3]. Although the soleus muscle is often overlooked, understanding its injury mechanisms is crucial for effective management and prevention of re-injury. Misdiagnosis remains a significant challenge, emphasizing the need for improved awareness and diagnostic techniques in sports medicine [16].

The relationship between muscle control, intrinsic joint movements, and foot load distribution is thought to have important clinical implications for Achilles tendon injuries [17]. The widths of connective tissues in the soleus provide a unique complexity of muscle fiber arrangements that may contribute to deformation patterns and subsequent susceptibility to injury. Although studies have shown no motor impairment between different morphologies and locations within the soleus muscle, the distinct characteristics of the connective tissues must be considered when understanding the structural anatomy and its relationship to muscle mechanics and injury. Awareness of these anatomical changes is also critical to understanding the changes in diagnostic imaging, ultimately supporting more accurate regional diagnoses and better defining appropriate treatment directions [18].

### 1.3. Analysis of Diagnostic Challenges in Soleus Muscle Injuries

Calf muscle injuries most commonly occur at the myofascial junction (MFJ) or the musculotendinous junction (MTJ), as these two sites represent a transition point between tissues with different elastic properties [10]. The analysis of diagnostic challenges in soleus muscle injuries (Table 1) reveals a complex interplay of anatomical, physiological, and methodological factors contributing to the difficulties in accurately identifying and treating this often-overlooked injury. Consequently, the effectiveness of palpation techniques and clinical tests used to assess calf injuries may need to be revised to diagnose soleus injuries accurately. This leads to a significant risk of underreporting and mismanagement, as the symptoms of dull, deep pain in the lower leg can often be mistaken for issues related to more superficial structures. Moreover, the reliance on subjective measures when assessing pain and mobility can result in variability in diagnosis, as individual pain thresholds and reporting can change based on factors such as psychological state or previous injuries. The latter can complicate the diagnostic process, as a history of injury can influence the clinician’s interpretation of symptoms and the patient’s pain reporting. Additionally, there needs to be more standardized methodologies specifically tailored to diagnose and evaluate soleus muscle injuries [18].

While advancements in imaging techniques such as magnetic resonance imaging (MRI) and ultrasonography hold promise for obtaining more precise diagnostic information, inconsistencies in how these modalities are applied further mar the validity of the findings. For example, while diffusion tensor imaging has emerged as a method to assess muscle architecture, its application to the soleus muscle still needs to be explored in clinical environments, making it challenging to implement comprehensive diagnostic protocols [19]. The challenge is compounded by the need for more well-defined criteria for categorizing the severity of soleus injuries. Current classifications primarily focus on more commonly injured muscles and do not specifically consider the anatomical and functional diversity of the soleus. This lack of clarity can lead practitioners to adopt generalized treatment approaches that fail to address the unique characteristics of soleus muscle injuries. As such, clinicians may resort to traditional management strategies—often borrowed from treating other calf muscles, freeing no attention to the specific demands of the soleus during athletic activities. Therefore, the lack of a clear diagnostic framework contributes significantly to treatment inefficacies [8].

Current diagnostic techniques for soleus muscle injuries primarily revolve around traditional imaging modalities, clinical assessments, and functional tests; however, these approaches often need to be revised to provide a comprehensive understanding of the injury’s nature and implications [11]. Magnetic resonance imaging (MRI) is commonly utilized because it allows for detailed soft tissue visualizations, including muscle and tendon structures. Despite its effectiveness in detecting significant tears or abnormalities, MRI can be limited in detecting subtle changes within the soleus muscle that may not translate to visible disruptions but affect muscle function [6].

Standard tests such as palpation and functional movement assessments are critical for establishing a preliminary diagnosis [4]. However, these assessments can be subjective and significantly influenced by the clinician’s experience and bias. Moreover, the subjective nature of these techniques often leads to varied inter-rater reliability, resulting in inconsistent diagnoses across different practitioners. Functional assessments may not comprehensively evaluate the muscle’s capacity and could overlook subtle deficits that reflect underlying injury severity or recovery needs. Consequently, while these conventional methods hold relevance, they often fail to provide a holistic view of the injury, which may ultimately compromise treatment efficacy. The increasing emphasis on the role of the fascial system in musculoskeletal health also highlights limitations in traditional diagnostic techniques [11]. Research indicates that injuries to the fascial system can contribute to performance detriments and lead to chronic pain conditions. Examining fascial and muscle dynamics often requires advanced imaging modalities and methodologies that still need to be widely adopted in clinical settings. Recent literature notes that a better understanding of the fascial system promises valuable injury prevention and treatment improvements [14]. Yet, current diagnostic practices generally need an integrated approach encompassing fascial assessments alongside traditional muscular evaluations. Furthermore, existing methods often need to account for the complex interactions between different muscle groups and their fascial connections, which can yield critical insights into injury mechanisms. Another key consideration in the current diagnostic landscape is the reliance on quantitative measures that may not accurately correlate with functional performance, particularly in populations such as athletes. Children with conditions such as cerebral palsy have been studied extensively regarding muscle hyperreflexia and its impact on functional mobility. This reveals a need for diagnostic methods considering functional and performance-related indicators [20]. The failure to integrate these performance-based assessments into the diagnostic process for soleus injuries can lead to an underappreciation of how these injuries impact overall functionality and athletic performance. The need for comprehensive and standardized protocols is further underscored by epidemiological research findings highlighting injury incidences and outcomes among athletes [21].

Both ultrasound and MRI are helpful tools in diagnosing posterior leg muscle injuries. Both help confirm the tear and explain its location within the muscle–tendon–bone unit. The deep area of the soleus muscle and its complex anatomy with many internal connections sometimes make it difficult to properly and thoroughly evaluate with ultrasound. MRI is a sensitive imaging modality in confirming soleus strains, and the sensitivity of USG is lower (27.2%) when comparing both methods [11]. However, from our point of view, the USG has several advantages that make it a valuable tool for the evaluation of soleus muscle injuries, considering that it is accessible, cost-effective, and offers the possibility of interviewing the patient during the examination to precisely define the painful site by squeezing it with the probe. Nevertheless, there is a strong correlation between USG sensitivity and the operator’s experience in terms of knowledge of muscle anatomy, interpretation of clinical data, and management [1]. However, when performed by trained professionals, the USG is reported to have good to excellent intra- and inter-rater reliability [22].

Balius et al. (2013) [11] reported that, after reviewing 55 cases, 24 cases (43.7%) had myofascial injuries on MRI, which were located in the posterior myofascial tendon (PMF) in 15 cases (27.3%) and the anterior myofascial tendon (AMF) in 9 cases (16.4%). Thirty-one cases (56.3%) were musculoskeletal injuries, of which nine cases (16.4%) were medial myofascial tendon (MMT), eleven cases (20%) were lateral myofascial tendon (LMT), and eleven cases (20%) were medial myofascial tendon (CMT). Compared with MRI, USG could detect soleus muscle injuries in 27.2% of cases. USG alone did not detect any injuries. The authors were more likely to detect posterior myofascial injuries than anterior myofascial injuries or all types of myofascial injuries. USG patterns for each type of injury have been described. The authors postulate that USG is not a more sensitive technique for detecting and assessing post-traumatic soleus tears compared with MRI. However, sensitivity increases with careful USG examination based on the specific anatomy of the muscle, in which small fibrous myofascial clusters and thinning of the fibrous area can be observed. The timing of the USG examination may be necessary for diagnosis [11].

Prakash et al. (2018) [2] reviewed 100 patients with clinically suspected and MRI-proven calf muscle injuries. Each injury was graded for the specific muscle involved, the location of the injury, and connective tissue integrity. Muscle tears were graded on a scale of 0 to 3, depending on muscle and connective tissue injury severity. Of the 100 patients, 63 had connective tissue involvement, with 18 being graded as grade 3, indicating severe injury. This highlights the prevalence of connective tissue injuries in calf muscle tears. The study found a significant correlation between the injury severity and the time athletes returned to play [2].

Connective tissue integrity conclusions: Connective tissue integrity is a valuable indicator for estimating recovery time after calf muscle tears. This finding could help develop treatment and rehabilitation strategies for athletes with such injuries. The study highlights the importance of assessing connective tissue integrity in calf muscle injuries, as it directly impacts recovery time and returns to sports [2].

Grade 0: injuries had an average recovery time of 8 days.

Grade 1: injuries took about 17 days.

Grade 2: injuries required about 25 days.

Grade 3: injuries had an average recovery time of 48 days.

Integrating evidence-based approaches into clinical pathways will be vital to redefining the diagnostic approach to soleus injuries, promoting better outcomes with more tailored treatment strategies. In addition, increasing awareness of the importance of the soleus in sports performance and rehabilitation may lead to the development of dedicated assessment tools that will allow clinicians to differentiate soleus injuries from those involving other muscle groups more effectively. In summary, the barriers to accurate diagnosis of soleus injuries are multifaceted and include anatomical challenges, lack of consensus on diagnostic criteria, limited access to advanced imaging technology, and sociocultural dynamics that influence athlete behavior.

**Table 1:** Physical examination and differential diagnosis.

Type of Damage	Symptoms	Additional Information
Soleus muscle	Pain is felt most in the posterolateral calf. Pain during passive ankle dorsiflexion or resisted ankle plantarflexion with the knee bent [4].	Ultrasound may not confirm morphological changes; it is necessary to perform MRI [6].
Tennis leg	Patients often report immediate, intense pain in the calf, typically during activities like running or jumping. Localized swelling and tenderness around the medial gastrocnemius muscle are joint, often accompanied by difficulty in weight-bearing. Increased pain during dorsiflexion of the foot, both actively and passively, is frequently observed, Clinicians may find palpable defects in the muscle, indicating the severity of the injury [6].	In this case, an ultrasound examination is an excellent diagnostic tool. MRI examinations are not always necessary [23].
Neuropathy	Tibial nerve Sensory Symptoms Patients often report abnormal sensations, such as burning or tingling, particularly in the sole of the foot and the medial malleolus. Pain: Chronic pain can occur, especially in the calf and sole, and is often exacerbated at night. Motor Symptoms Weakness: There may be noticeable weakness in foot flexors, leading to difficulty in plantar flexion and inversion of the ankle Muscle Atrophy: Prolonged nerve damage can result in muscle denervation and atrophy in the affected areas [24] Personal nerve Inability to bend the foot in the dorsum of the foot, difficulty bending the toes. Bird gait (bending the leg at the knee and lifting it, placing the foot on the toes, then on the side of the foot, and finally on the heel [25].	The most common findings in pathologies of the peroneal nerve were hypoechoic thickenings. In inflammation, the extended segment of the nerve is affected by edema, and ultrasound examination shows hypoechoic thickening of the involved segment of the nerve, with loss of intraneural hypoechoic tissue peripherally surrounded by interfascicular epineurium. On ultrasound examination, small fascicles may appear as a single undifferentiated structure and indistinguishable from the surrounding interfascicular epineurium or a cluster of fascicles. Electrodiagnostic testing is essential for identifying the type and severity of neuropathy [25].
Achilles tendon	Performing palpation tests and muscle power tests with Thompson’s sign and Matles’ test [17].	High-resolution ultrasonography is effective in diagnosing Achilles tendon lesions. It shows comparable results to MRI for tendinopathy and full-thickness tears while excelling in early enthesitis detection [26].
Venous diseases	Cyanosis occurs, Homans and Mayr symptoms, we perform the Wells scale and Virchow’s triad [27].	The basic examination is a power Doppler of the veins, which can confirm venous thrombosis and a blood d-dimer test. It should be remembered that d-dimers are not specific markers and may also be elevated, for example, in injuries and inflammations [27].
Arterial diseases	Intermittent claudication and pain occur, which are worse on walking and subside with rest. There may be abnormal peripheral pulses and arterial bruits [28].	We examine using Doppler examination, ankle-brachial index, and angiography [28].

### 1.4. Case Report

Anamnesis: The patient (39 years old, BMI: 22.2 training experience: 22 years) reported to the Provita Medical Center about pain imitating symptoms from the Achilles tendon. The man felt pain around the Achilles tendon at the height of the medial malleolus. He played amateur football twice a week. In his youth, he played football regularly, playing in a club. He had not previously had any injuries to the calf muscles or Achilles tendon. The pain was slight, only exertional, without symptoms of swelling or hematoma. The ultrasound examination showed no morphological changes, so a 4-week rest was recommended, with exercises recommended according to the modified Alfredson protocol and a return to play. Unfortunately, after six weeks, the man returned with intensified symptoms, and the ultrasound examination performed showed damage to the distal tendon–muscle junction. This case confirms the diagnostic difficulties with using ultrasound in the first symptoms of the soleus muscle and recurrent injury due to this.

### 1.5. Imaging Tests

**Figure 1:**
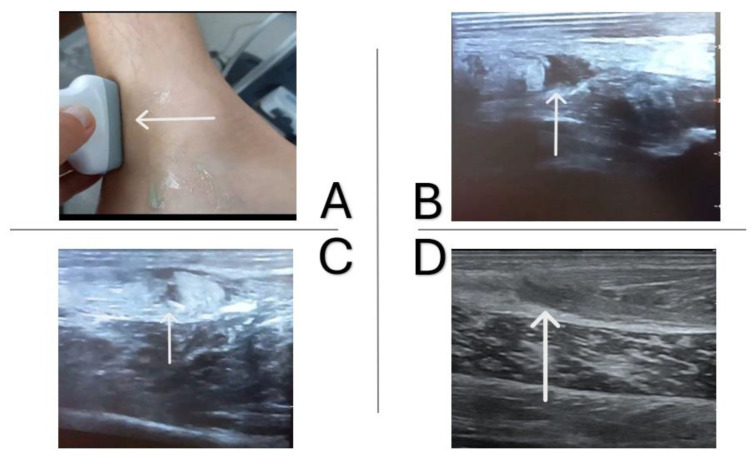
Ultrasound examination of the soleus muscle (**A**,**B**) immediately after the injury, (**C**) after 8 weeks of treatment, and (**D**) after 16 weeks of treatment.

**Figure 2:**
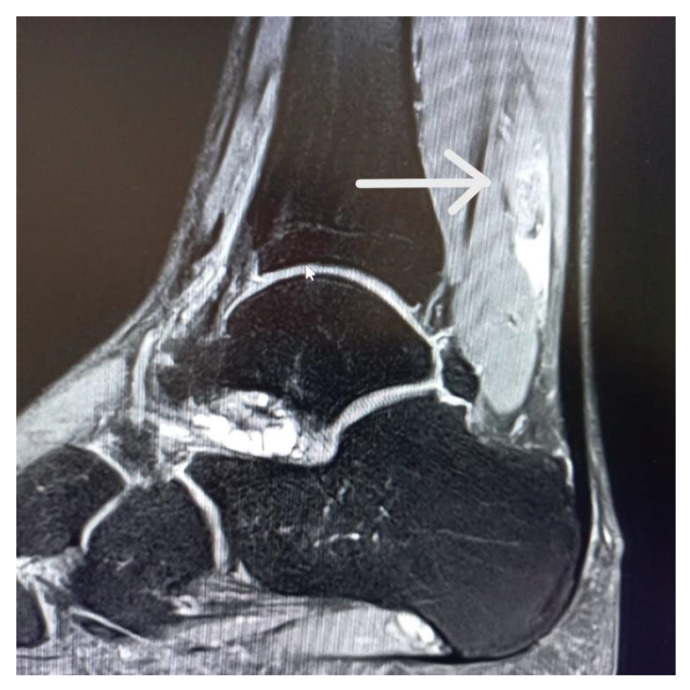
MRI scan 2 weeks after injury.

The beginning of the patient’s imaging diagnostics was an ultrasound examination, which initially did not show any significant morphological changes, in correlation with the clinical examination and symptoms of only discomfort after a 4-week break and kinesitherapy, he was allowed to play. After another 2 weeks, a severe injury occurred during the game, and the ultrasound showed a significant degree of damage (Figure 1A,B). Next, after the patient was provided with care, an MRI examination was ordered, which the patient performed after another 2 weeks and confirmed the previous diagnosis (Figure 2). The following examination was performed 8 weeks after the injury, the next one after 16 weeks after the injury (Figure 1C,D).

## 2. Physiotherapy Protocol

**Figure 3:**
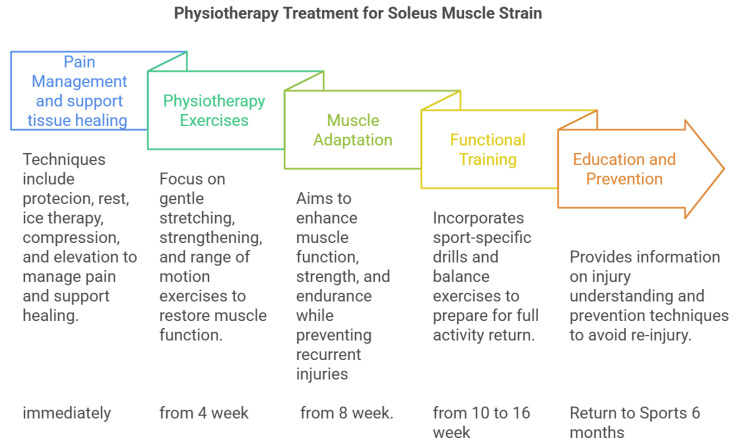
General model of physiotherapy after muscle injury.

The role of physiotherapy after soleus muscle injury is leading to the consideration of conservative treatment. An essential element of this strategy is the use of a rational scheme to introduce individual physiotherapy methods based on the “biological clock” of healing (Table 2) of tendon tissues (Figure 3).

In the immediate aftermath of a soleus injury, the PRICE approach [29]—consisting of tissue protection (walker), rest, ice, compression, and elevation—is a critical initial approach to facilitating recovery and mitigating further damage [30]. Rest allows the injured muscle to heal by minimizing strain and preventing exacerbation of the injury, while ice application effectively reduces inflammatory responses and relieves pain [10]. Compression serves to limit swelling and provide support to the affected area, improving circulation and facilitating healing. Elevation is a natural response and helps reduce pain and swelling in the initial stages of injury by promoting venous return from the injured site. The culmination of these practices creates a supportive environment for recovery and provides a foundation for subsequent physical therapy strategies [29]. Using game-ready cryocompression therapy [31,32] at this stage seems particularly important and practical. The patient used game-ready therapy ×3 daily for 14 days, 10 min per session.

Following a soleus injury, implementing isometric exercises almost immediately after injury, followed by concentric–eccentric exercises 7 days after injury with ankle restriction for up to 3 weeks after injury to protect the developing scar, is a critical strategy to facilitate recovery [23]. These exercises reduce muscle arthrosis, maintain restricted mobility, and prevent ankle stiffness, promoting functional movement patterns necessary for daily activities and athletic performance [18]. In our case, at this stage, we recommend incorporating blood flow restriction training to enhance the rehabilitation process by minimizing disuse atrophy while allowing controlled exercise, which benefits healing and strength restoration [33].

**Figure 4:**
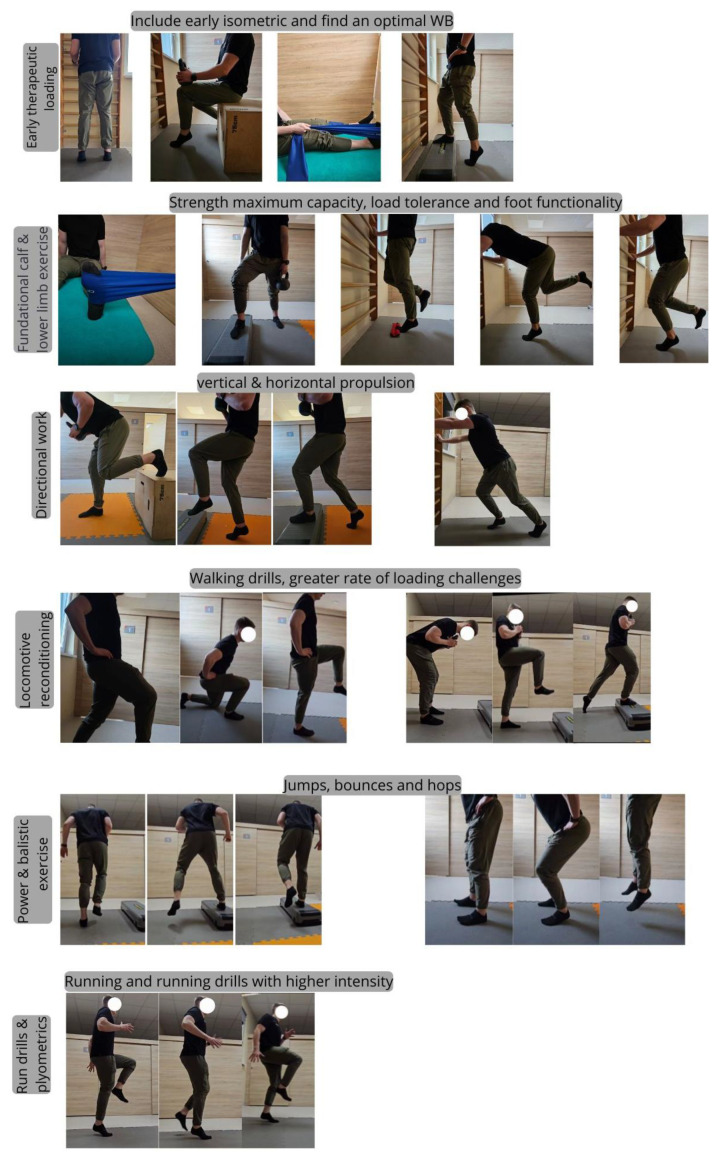
Kinesiotherapy scheme taking into account the individual stages of tissue healing.

Incorporating progressive strengthening and conditioning (Figure 4) into physical therapy protocols for soleus injuries is key to restoring function and adaptation [4]. This systematic approach emphasizes progressively increasing exercise intensity and complexity, tempo of movement, and movement patterns, facilitating muscle healing and functional recovery [34]. Because the soleus contributes significantly to activities such as walking and running, customized strengthening programs address muscle imbalances and improve overall kinetic chain efficiency [18]. Visual guidance, such as recording the patient’s movement pattern, can further support the implementation of effective progressive training protocols, and we encourage physical therapists to do so.

Effective treatment of muscle injuries requires a comprehensive approach synthesizing various methods, including those from physical medicine. Still, the comparative effect of these different treatment methods remains insufficiently studied. Physiotherapists have a full range of therapies, which they use depending on the stage of tissue healing and their conviction in its use. While laser biostimulation has gained significant recognition in supporting the healing of muscle and tendon tissues and modulating pain at each stage [35], muscle electrostimulation plays a key role in the early healing, when the patient has limited functional possibilities and in the punctate stage to enhance the effect of training [36].

Laser biostimulation increases cell metabolism, enzyme activity, and endorphin release, which are crucial for tissue repair and regeneration [37]. It reduces the expression of inflammatory markers such as COX-2, thus reducing inflammation and facilitating tissue repair processes [38].

Another type of physical medicine is Tecar therapy [39]. Tecar works by delivering radiofrequency energy through capacitive and resistive electrodes that generate heat in the tissues to improve recovery outcomes. Tecar can also accelerate tissue repair and improve capillary permeability, leading to better inflammation management and pain modulation [40].

Electromagnetic fields (EMFs), especially pulsed electromagnetic fields (PEMFs), have emerged as a promising noninvasive treatment for muscle injuries and other musculoskeletal conditions. These fields enhance the body’s natural repair processes by influencing bioelectric fields at the cellular and tissue levels. Studies indicate that EMFs can reduce pain, improve muscle function, and speed recovery in musculoskeletal disorders [41].

Platelet-rich plasma (PRP) therapy has emerged as a promising treatment for muscle injuries, particularly in sports medicine [42]. In the case described, 3 mL of ultrasound-guided PRP was administered twice at 3 and 8 weeks post-injury.

PRP is believed to accelerate tissue regeneration and facilitate a faster return to physical activity by utilizing its regenerative properties. However, the efficacy of PRP may vary depending on preparation protocols and individual patient characteristics. Despite its potential, questions remain regarding its long-term effectiveness and optimal application conditions [43]. Preclinical studies suggest that PRP improves the healing process by promoting myoblast proliferation at the injury site. However, it may also enhance the catabolic phase, potentially prolonging healing time if too many leukocytes are in the preparation. Therefore, obtaining PRP is important [43]. The timing and content of PRP injections are critical because platelets contain profibrotic factors that can lead to fibrosis rather than muscle regeneration [44]. Although PRP therapy is a promising alternative for treating muscle injuries, its use is not without challenges. The potential for fibrosis and the need for precise timing and formulation underscore the complexity of effectively using PRP. Furthermore, comparisons with PPP suggest that alternative plasma treatments may offer competitive or superior results in some scenarios, warranting further study and high-level research to determine the most effective treatment protocols [43].

### Principles of Load Selection Based on Objective Data

**Figure 5:**
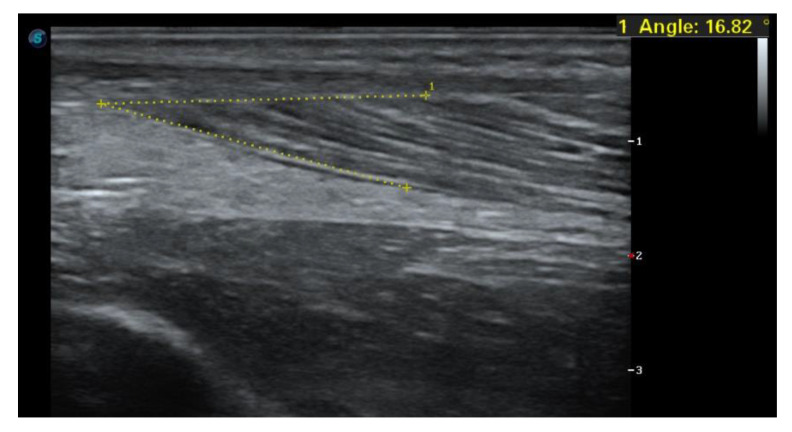
Pennation angle of the flat muscle on the healthy side of the patient at rest.

Assessment of the angle of pennation (inclination) (Figure 5), functional testing, and muscle strength assessment are critical to determining an athlete’s readiness to return to physical activity after injury and the amount of loading applied in physical therapy. These metrics provide insight into functional recovery and the potential risk of re-injury [45]. Muscle strength testing, mainly through isokinetic assessments, provides a reliable measure of an athlete’s recovery and readiness for sport-specific exercises. Integrating these tests into physiotherapy protocols can improve safe return to sport decision-making. The inclination angle (i.e., the angle at which muscle fibers are positioned relative to the tendon line) is a key determinant of muscle strength because it affects the placement of muscle fibers relative to the axis of force generation. Increasing the inclination angle allows more fibers to be packed in parallel, increasing the ability to generate force. Research indicates that an inclination angle of up to 45 degrees significantly increases the force generated in the direction of the muscle fibers and the component of force transferred to the tendon, thus influencing overall muscle strength [46]. Ultrasound assessment of the angle of pennation and muscle strength plays a key role in assessing an athlete’s readiness to return to physical activity after injury. The angle of pennation increases as muscles adapt to loading, allowing the muscle to generate greater force. After the injury, a decrease in the pension angle may indicate atrophy or inadequate muscle recovery. We recommend comparing the pennation angle in maximal isometric contraction with the healthy side, and deviations of more than 10% may pose a risk of re-injury [47].

Isokinetic dynamometers are widely used to assess muscle strength. They provide peak torque values and allow the assessment of muscle groups throughout the range of joint motion. This method is particularly beneficial in physiotherapy settings due to its safety and ability to quantify muscle strength accurately [48]. Dynamometers are a more straightforward solution. Due to their cost and availability, they can be used in any physiotherapy clinic and training setting. In this case, the Kinvent Force Dynamometer is a key tool for assessing muscle strength, particularly on the quadriceps [49]. Utilizing advanced technology, this dynamometer accurately measures isometric strength, an essential element in assessing athletic performance in various sports. The device has been validated for reliability and demonstrates excellent intra- and inter-rater consistency, critical to ensuring accurate assessments in clinical and sports settings [50]. Furthermore, research reveals that Kinvent can effectively differentiate athletes from different sports, reflecting differences in muscle strength attributable to specific training regimens [51]. Furthermore, its portability and user-friendly design facilitate its use in various environments, increasing its utility in rehabilitation contexts. Overall, the Kinvent Force Dynamometer represents a significant advance in sports science and rehabilitation, contributing to our understanding of force metrics and their impact on performance optimization [52].

## 3. Discussion

This literature review aims to summarize the current scientific knowledge about the diagnosis and treatment of soleus muscle injuries. Combining it with a case study report and the authors’ own experience, it aimed to formulate recommendations for differential diagnosis methods, treatment strategies, and future research directions.

The treatment of soleus injuries has evolved considerably but is still plagued by inconsistencies and gaps in evidence-based practice. Current treatment methods can be broadly divided into nonoperative and operative strategies, each with potential benefits and limitations. Nonoperative approaches often emphasize conservative treatment techniques such as protection, rest, ice, compression, elevation (PRICE protocol), tissue healing support, and physical therapy exercises to restore flexibility, strength, and proprioception [4]. However, the variability in the effectiveness of these techniques is still a matter of debate in the literature, suggesting the need for further rigorous research to develop a standardized treatment framework. Furthermore, evidence suggests improving muscle performance through targeted strengthening exercises may benefit athletes recovering from soleus injuries. Implementing eccentric training—specifically focused on lengthening the muscle under tension—has shown promising results in preventing relapses and promoting recovery in other muscle groups. This approach may also be beneficial, but empirical support specifically targeting this muscle is limited, and most of the current knowledge comes from broader studies of calf muscle injuries [6].

Physiotherapy methods give the highest level of recommendation for tendon and muscle injuries [18]. However, research is still needed on increasing training, movement tempo, movement patterns, and the amount of load applied during the healing phases of the injured muscle. Some results suggest positive results in the reinnervation of the injured muscle. Moderate daily exercise performed on a treadmill level causes increased axonal regeneration and slightly improved functional recovery in rats [53]. If exercise is performed on an inclined treadmill, even more axons are effectively regenerated, causing a more significant improvement in functional recovery. However, it emphasizes the effectiveness of different exercise protocols, especially intermittent treadmill exercise of moderate intensity, in improving muscle innervation and functional recovery after nerve injuries [7].

Exercises give the highest level of recommendation for tendon and muscle injuries. However, research is still needed on increasing training, movement tempo, movement patterns, and the amount of load applied during the healing phases of the injured muscle. Some results suggest positive results in the reinnervation of the injured muscle. Moderate daily exercise performed on a treadmill level causes increased axonal regeneration and slightly improved functional recovery in rats [54]. If exercise is performed on an inclined treadmill, even more axons are effectively regenerated, causing a more significant improvement in functional recovery. However, it emphasizes the effectiveness of different exercise protocols, especially intermittent treadmill exercise of moderate intensity, in improving muscle innervation and functional recovery after nerve injuries [55].

In some cases where conservative treatment fails, surgical intervention becomes an option, although it is often considered a last resort. Surgical techniques for soleus injuries may involve debridement or repair of torn muscle tissue and usually require comprehensive pre- and postoperative rehabilitation protocols to maximize results. However, surgical outcomes are highly variable and often complicated by factors such as the severity of the initial injury and patient compliance with rehabilitation protocols. The complexity of treatment decisions is compounded by the current literature, which needs a more in-depth comparative analysis of the effectiveness of surgical and nonsurgical treatments. More robust research would be invaluable in defining the patient population best served by each modality [56].

Research is also emerging into the nonstructural components of treatment, particularly the role of psychological factors in recovery. Understanding the mental health aspect of recovery can increase adherence to physical rehabilitation programs and ultimately impact outcomes. Techniques such as cognitive behavioral therapy, visualization, and mindfulness have been integrated into rehabilitation practices to address any psychological barriers to recovery. This holistic approach emphasizes the need to recognize the multifaceted nature of musculoskeletal injuries, in which physical recovery is closely linked to emotional well-being [57].

Diet plays a crucial role in recovery from muscle injury, and specific nutrients and dietary strategies enhance the healing process. Integrating bioactive peptides, proteins, antioxidants, omega-3 fatty acids, and probiotics can significantly aid muscle repair and regeneration. These nutrients work through various physiological pathways to reduce inflammation, support muscle protein synthesis, and improve overall recovery outcomes [58]. Bioactive peptides from food viruses can be introduced through the cellular network and promote tissue adaptations, meeting pathways such as PI3K/Akt/mTOR and NF-κB, which are crucial for cellular certifications [59]. Antioxidants help manage oxidative stress during the inflammatory phase of muscle damage. Omega-3 fatty acids support anti-inflammatory pathways and increase anabolic sensitivity, helping to repair muscle [60]. Quercetine is a flavonol-type polyphenol and Zynamite^®^ (mango leaf extract) is a natural polyphenol; both have antioxidant and anti-inflammatory attributes that may stop Exercise Induced Muscle Pain (EIMD) and promote muscle recovery [61,62]. Adequate protein intake is essential, with recommendations ranging from 1.6 to 2.5 g/kg/day, spread between meals, to maintain muscle mass and counteract anabolic resistance during the repair phase [58]. Nutritional supplementation—focusing on critical nutrients such as collagen, amino acids, and specific antioxidants—can accelerate healing by enhancing cellular repair processes. Some research shows that an inappropriate diet can have a negative impact and increase EIMD [63]. Research by Nieman et al. and Saracino et al. shows the positive impact of protein consumption on reducing muscle damage and inflammation, underscoring that the quality and quantity of the proteins play an important role [64,65]. This association underscores the need for interdisciplinary approaches to treating soleus injuries, combining insights from sports medicine, nutrition, and physical therapy to create comprehensive rehabilitation protocols [58].

Research into task-dependent and biofeedback-assisted physical therapy approaches reveals promising avenues for improvement. Studies have shown that while conventional physical therapy approaches are unable to comprehensively address muscle activation patterns, techniques that incorporate feedback mechanisms have the potential to significantly improve rehabilitation efficacy. EMG assessment identifies specific activation deficits in the soleus muscle that can be targeted with individualized exercise regimens. This improves functional outcomes during activities requiring ankle plantar flexion and overall lower limb stability [66].

Ultrasound-guided percutaneous needle electrolysis (PNE) combined with an eccentric exercise program is a practical physical therapy approach for treating chronic soleus injuries. This method improves pain relief, ankle dorsiflexion, range of motion, and function. Eccentric exercises specifically target the plantar muscle, promoting recovery through controlled loading. Research indicates that this combination treatment produces better results than either intervention alone, making it a valuable therapeutic tool for dancers suffering from chronic soleus injuries [67].

Both myofascial release and pressure release techniques effectively reduce pain and increase ankle dorsiflexion range of motion in individuals with soleus myofascial trigger points. These manual therapy techniques provide similar benefits, making them viable treatment options for soleus myofascial trigger points. Maintaining the soleus muscle at an extended length during contraction may prevent initial muscle injury and reduce subsequent damage. This approach minimizes excessive sarcomere contraction and other structural changes, suggesting a protective effect against muscle injury [68]. Vibration therapy and radial pressure waves are also proposed in the treatment of soleus muscle injuries, reducing excessive post-traumatic muscle stiffness [30].

## 4. Limitations of the Current Studies and Future Research Directions

Many studies published in scientific journals have limitations such as accessibility, variability in treatment protocols, and lack of standardization in the application of techniques. The lack of direct comparison between surgical and nonsurgical treatments is an issue that needs explanation. However, it would be challenging to propose possible methods to compare these treatments, such as more controlled studies, multicenter clinical trials, or a systematic review of the literature to summarize the current scientific knowledge.

Considering the findings regarding the diagnostic and treatment challenges associated with soleus muscle injuries, future research directions are crucial to shaping effective interventions and improving outcomes for individuals affected by injury. Exploring soleus muscle injuries’ biomechanical and physiological intricacies should be a priority, mainly through advanced imaging techniques such as high-resolution ultrasound and MRI, which have shown promising results in visualizing muscle architecture and elastic properties in various contexts. Current treatments often need more detail regarding the regeneration of this muscle, and the development of evidence-based clinical practice guidelines based on comprehensive research findings would fill this critical gap. Future studies should be designed to evaluate the effectiveness of various interventions, including mechanotherapy, eccentric training, and neuromuscular electrical stimulation, particularly regarding patient-specific factors such as age, activity level, and pre-existing injuries.

## 5. Conclusions

The pain in the calves should not be ignored because no intervention increases the risk of acute injury or developing an overuse injury. A precise differential diagnosis is crucial for choosing adequate treatment methods that may decide on their effectiveness. The MRI and USG methods are reliable and useful tools for the diagnosis of soleus muscle injury. In practice, after choosing the treatment strategy, the important factor is constant monitoring of the morphological ability to regenerate muscle to tolerate loads at every stage of physiotherapy and the effects of the intervention. More research is needed to provide knowledge leading to the design of evidence-based protocols for the treatment of soleus muscle injuries.

## Figures and Tables

**Table 2:** Potential healing time depending on the type of injury.

Injury Type	Prevalence (%)	Common Cause	Typical Recovery Time (Weeks)
Soleus Strain	15	Overuse or sudden increase in activity	2
Soleus Tear	10	Trauma or high-force contraction	4
Soleus Tendinopathy	5	Repetitive stress and improper biomechanics	6
Partial Tear	8	Acute injury with swelling	3
Complete Tear	2	Severe trauma or sudden excessive force	12

## Data Availability

No new data was generated for this article.
